# Resolution Threshold of Current Molecular Epidemiology of Diphtheria

**DOI:** 10.3201/eid2011.140094

**Published:** 2014-11

**Authors:** Igor Mokrousov

**Affiliations:** St. Petersburg Pasteur Institute, St. Petersburg, Russia

**Keywords:** diphtheria, Corynebacterium diphtheriae, bacteria, genotyping, genomics, molecular epidemiology, clusters, whole-genome sequencing, pulsed-field gel electrophoresis, multilocus sequence typing, regularly interspaced short palindromic repeats

“The fox who longed for grapes, beholds with pain

The tempting clusters were too high to gain;

Grieved in his heart he forced a careless smile,

And cried, ‘They’re sharp and hardly worth my while.”

(Aphra Behn, 1687, after Aesop’s *The Fox and the Grapes*)

**To the Editor:** Diphtheria is an extremely rare disease in Europe but remains a major health issue in developing countries (*1**–**3*). In recent years, steady progress has been made toward understanding the factors of pathogenicity of its causative agent (*Corynebacterium diphtheriae*). In contrast, remarkable advances in its basic genomics have not been sufficiently translated into the molecular epidemiology of diphtheria. A recent report by Zasada (*4*) offers an apt opportunity to take a new look at this issue.

The current genotyping repertoire of *C. diphtheriae* includes several methods but those most frequently used are classical ribotyping and pulsed-field gel electrophoresis (PFGE). More recently, a multilocus sequence typing (MLST) scheme for *C. diphtheriae* was developed (*5*). Compared with ribotyping, PFGE, and other methods based on analysis of banding profiles, MLST results are digital, unambiguous, and portable. MLST discrimination of 150 isolates from 18 countries and spanning 50 years was “in accordance with previous ribotyping data, and clonal complexes associated with disease outbreaks were clearly identified by MLST” (*5*).

In the report by Zasada (*4*), all 3 recommended methods (PFGE, MLST, and ribotyping) were used to genotype 25 nontoxigenic *C. diphtheriae* isolates from Poland. The author concluded that these isolates “represent a single clone despite isolation … in different part of the country over a 9-year period” and raised the question of whether a single clone of *C. diphtheriae* is circulating in Poland (*4*). These isolates are related genetically, but do they represent a truly single clone or might they be further discriminated? Their circulation in Poland may be caused by their high pathogenicity, but also (or instead) it might reflect their endemic, historical prevalence in this country. I believe that these questions are unlikely to be answered by the internationally agreed-upon methods for *C. diphtheriae* typing because of their insufficient resolution: the discriminatory power of MLST does not exceed to that of ribotyping (*5*).

Two in silico–inspired approaches have recently been pursued toward more precise molecular genetics and epidemiology of diphtheria. The first approach is based on whole-genome sequencing (WGS). After years of stagnation, the number of complete *C. diphtheriae* genomes has finally started to increase: currently 13 complete and 3 draft genomes are available in GenBank (as of April 14, 2014). The second approach is locus oriented and makes use of the repetitive DNA sequences, namely, variable number tandem repeats (VNTR) and clustered, regularly interspaced short palindromic repeats (CRISPR) loci.

A study in Poland showed a discriminatory capacity of some of the VNTR loci in *C. diphtheriae* (*6*), although preliminary results were not compared with those from other typing methods. CRISPR loci in *C. diphtheriae* have been studied in more detail and CRISPR-based spoligotyping showed a high level of discrimination for an epidemic clone in Russia and Belarus (*7**,**8*). In particular, 156 isolates from Russia of the epidemic clone (classical ribotypes Sankt-Petersburg and Rossija) were subdivided into 45 spoligotypes (*7*).

Further studies underlined the limitations of CRISPR-based typing: the 3 described CRISPR loci are not present simultaneously in all isolates; and most strains have unique spacers at the leader part of the array, which indicates their independent evolution after they diverged from a common ancestor (*9*). Accordingly, Sangal et al. suggested that CRISPR-based typing might not necessarily provide information on evolutionary relationships between different strains, but it might offer a high level of discrimination to study local epidemiology (*9*).

Recent advances in *C. diphtheriae* genomics concern an increasing number of complete genomes in GenBank, development of new ideas (e.g., revisiting biochemical subdivision into biovars) (*10*), and development of new typing schemes (MLST, VNTR, and spoligotyping). Nevertheless, in spite of lack of genetic support, biochemical classification into biovars is still uncritically used. In spite of other demonstrated or potential tools (e.g., CRISPR, VNTR), classical or new methods (all with limited resolution) are still used in many studies, both global and local. In spite of many available complete genomes, this wealth of information has not been translated into a WGS-informed high-resolution typing scheme. It might be sufficient to sequence all 3 CRISPR/*cas* loci in all strains studied by Zasada (*4*) to gain some insight into their relatedness and possible spatiotemporal evolution. A phylogenetically more robust, albeit more expensive, solution would be using WGS analysis to achieve the same objective.

In conclusion, molecular epidemiology of diphtheria would definitely benefit from implementation of more precise molecular genetics. First, WGS (or at least core genome) analysis might offer a broader range of possible general solutions from global tracing of large clonal clusters (current threshold) toward fine-tuned strain discrimination. At the same time, a multicenter evaluation of recently developed inexpensive and discriminatory VNTR and CRISPR methods is warranted to see if and how they could complement regional surveillance.
